# Supplementary material for: Identification of photoperiod-induced specific miRNAs in the adrenal glands of Sunite sheep (Ovis aries)
Source: Front Vet Sci. 2022 Jul 22;9:888207. doi: 10.3389/fvets.2022.888207 (PMC9354845; doi:10.3389/fvets.2022.888207)
Supplement: Supplementary file 1 [file Data_Sheet_1.ZIP › Supplementary file/miRDeep2 file.zip!/miRDeep2 file/Know_Structure/LP42A2/oar-mir-409.pdf]

```
miRBase precursor           : oar-mir-409
Total read count            : 11657
oar-miR-409-5p read count   : 1931
oar-miR-409-3p read count   : 9716
remaining reads             : 10
```

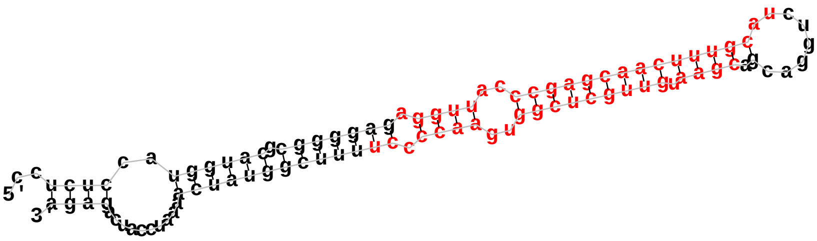

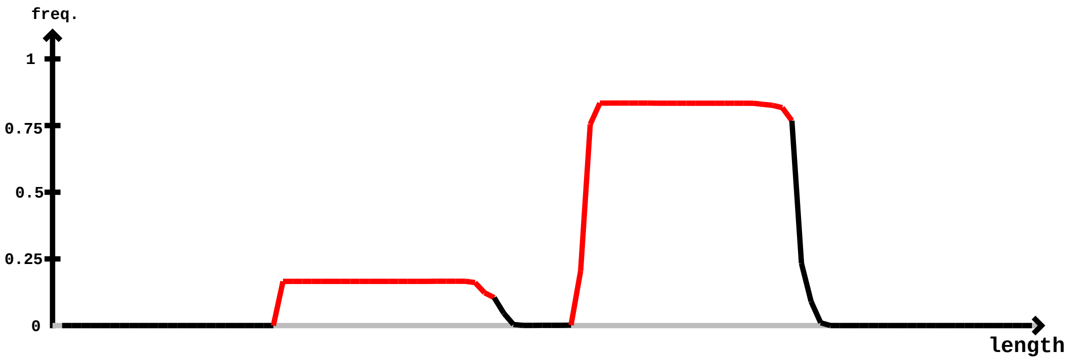


```
                        oar-miR-409-5p
                                            oar-miR-409-3p
5'- ccucuccaugguacgcggggagagguuacccgagcaacuuugcaucuggacgacgaauguugcucggugaaccccuuuucgguaucaaaauccaucagaga  -3'  exp
    .((((...((((((.(((((((.((((..((((((((((((((........).)))).)))))))))..)))).)))))))))))))).........)))).  reads  mm  sample
    ......................agguuacccgagcaacuuu............................................................  2     0   seq
    ......................agguuacccgagcaacuuug...........................................................  56    0   seq
    ......................agguuacccgagcaacuuugc..........................................................  445   0   seq
    ......................agguuacccgagcaacuuugca.........................................................  214   0   seq
    ......................agguuacccgagcaacuuugcau........................................................  684   0   seq
    ......................agguuacccgagcaacuuugcauc.......................................................  488   0   seq
    ......................agguuacccgagcaacuuugcaucu......................................................  41    0   seq
    .......................gguuacccgagcaacuuugc..........................................................  1     0   seq
    ......................................uuugcaucuggacgacgaaugu.........................................  1     0   seq
    ......................................uuugcaucuggacgacgaauguu........................................  5     0   seq
    ............................................ucuggacgacgaauguugcu.....................................  1     0   seq
    .................................................acgacgaauguugcucggugaacc............................  3     0   seq
    ....................................................acgaauguugcucggugaac.............................  8     0   seq
    ....................................................acgaauguugcucggugaacccc..........................  1     0   seq
    .....................................................cgaauguugcucgguga...............................  1     0   seq
    .....................................................cgaauguugcucggugaa..............................  2     0   seq
    .....................................................cgaauguugcucggugaac.............................  32    0   seq
    .....................................................cgaauguugcucggugaacc............................  26    0   seq
    .....................................................cgaauguugcucggugaaccc...........................  35    0   seq
    .....................................................cgaauguugcucggugaacccc..........................  197   0   seq
    .....................................................cgaauguugcucggugaaccccu.........................  1487  0   seq
    .....................................................cgaauguugcucggugaaccccuu........................  428   0   seq
    .....................................................cgaauguugcucggugaaccccuuu.......................  152   0   seq
    .....................................................cgaauguugcucggugaaccccuuuu......................  14    0   seq
    ......................................................gaauguugcucggugaac.............................  7     0   seq
    ......................................................gaauguugcucggugaacc............................  15    0   seq
    ......................................................gaauguugcucggugaaccc...........................  57    0   seq
    ......................................................gaauguugcucggugaacccc..........................  328   0   seq
    ......................................................gaauguugcucggugaaccccu.........................  4092  0   seq
    ......................................................gaauguugcucggugaaccccuu........................  1111  0   seq
    ......................................................gaauguugcucggugaaccccuuu.......................  697   0   seq
    ......................................................gaauguugcucggugaaccccuuuu......................  96    0   seq
    ......................................................gaauguugcucggugaaccccuuuuc.....................  1     0   seq
    .......................................................aauguugcucggugaaccc...........................  9     0   seq
```

```
                      oar-miR-409-5p
                                                     oar-miR-409-3p
ccucuccaugguacgcggggagagguuacccgagcaacuuugcaucuggacgacgaauguugcucggugaaccccuuuucgguaucaaaauccaucagaga

.......................................................aauguugcucggugaacccc..........................    43     0     seq
.......................................................aauguugcucggugaaccccu.........................    660    0     seq
.......................................................aauguugcucggugaaccccuu........................    122    0     seq
.......................................................aauguugcucggugaaccccuuu.......................    79     0     seq
.......................................................aauguugcucggugaaccccuuuu......................    13     0     seq
........................................................auguugcucggugaaccccu.........................    1      0     seq
.........................................................uguugcucggugaaccccuuu.......................    1      0     seq
..........................................................guugcucggugaaccccu.........................    1      0     seq
```
